# The Use of Smart Speakers in Care Home Residents: Implementation Study

**DOI:** 10.2196/26767

**Published:** 2021-12-20

**Authors:** Katie J Edwards, Ray B Jones, Deborah Shenton, Toni Page, Inocencio Maramba, Alison Warren, Fiona Fraser, Tanja Križaj, Tristan Coombe, Hazel Cowls, Arunangsu Chatterjee

**Affiliations:** 1 Centre for Health Technology University of Plymouth Plymouth United Kingdom; 2 School of Health Professions University of Plymouth Plymouth United Kingdom; 3 School of Nursing and Midwifery University of Plymouth Truro United Kingdom

**Keywords:** voice-activated technology, smart speaker, care home, technology-enabled care, older people, learning disability, digital technology, consumer device, smart device

## Abstract

**Background:**

The use of smart speakers to improve well-being had been trialed in social care by others; however, we were not aware of their implementation in most care homes across a region in the Southwest of the United Kingdom. For the widespread adoption of new technology, it must be locally demonstrable and become normalized.

**Objective:**

The aim of this study was to install smart speakers in care homes in a rural and coastal region and to explore if and how the devices were being used, the barriers to their implementation, and their potential benefits.

**Methods:**

Email, workshops, drop-in sessions, phone, and cold calling was used to contact all 230 care homes, offering a free smart speaker and some advisory support. Care homes accepting the devices were asked to complete a feedback diary. Nonresponse rate for diary completion was high and was thus supplemented with a telephone survey.

**Results:**

Over the course of 7 months, we installed 156 devices in 92 care homes for older people, 50 devices for people with physical or mental health needs, and 8 for others. The devices were used mainly for music but also for poetry, recipes, light controls, jokes, and video calls. Care home managers reported the benefits for the residents, including enhanced engagement with home activities, enjoyment, calming effects, and the acquisition of new skills. Implementation problems included internet connectivity, staff capacity, and skills.

**Conclusions:**

Affordable consumer devices such as smart speakers should be installed in all care homes to benefit residents. Voice-activated technologies are easy to use and promote interaction. This study indicates that implementation in care homes was possible and that smart speakers had multifaceted benefits for residents and staff. Most care homes in this region now use smart speakers for their residents, thereby normalizing this practice.

## Introduction

The United Kingdom currently has 425,000 people requiring some form of residential care in over 11,000 care homes [1]. In Cornwall, two-thirds of care homes are for older people and one-third for people requiring physical and mental health care. Care homes are under pressure from an ageing population, an underresourced workforce [2], and currently from the impact of COVID-19.

Both the Social Care Institute for Excellence and the Care Quality Commission recognize the importance of technology in providing high quality care services [3,4]. For example, technology can help address loneliness through internet training and video call interventions [5].

Many projects have focused on cutting edge technologies still under development [6] rather than commercially available products that may improve quality of life now in residential care. Exceptions include the use of affordable companion robots [7]. Implementing technology use such as Skype (Microsoft Inc) in care homes has not always been straightforward [8]. Studies of smart home technology have identified desired features such as emergency help, health monitoring, and environmental control [9,10]; however, their implementation may be prevented due to cost [11].

The literature has numerous examples of “pilots” and small-scale studies and national programmes, such as the United Kingdom “delivering assisted living lifestyles at scale” project [12,13], which were set up to try to address implementation at scale conceptual frameworks on technology implementation such as Greenhalgh’s Non-adoption, Abandonment, Scale-up, Spread, Sustatinability framework [14], May and Mair’s Normalization Process Theory (NPT) [15], the longer standing Technology Acceptance Model [16] and the Unified Theory of Acceptance and Use of Technolgy [17], and the original work of Roger [18] all include the idea of demonstrability at scale and reaching critical mass [19]. Although the Unified Theory of Acceptance and Use of Technology 2 framework has previously been used to examine the adoption of voice-activated digital assistants with community-dwelling older adults [20], we find the NPT framework to be more appropriate for assessing and enhancing the implementation of complex interventions into routine practice, the process known as normalization. The 4 NPT components for the successful integration of interventions are coherence, cognitive participation, collective action, and reflexive monitoring.

Smart speakers became commercially available in 2014 [21] and have seen a rapid uptake with 20% of households now owning one [22]. In 2018, those aged 55 or older comprised 33% of smart speaker ownership, while 14- to 18-year-olds comprised only 10% [23]. Smart speakers are currently available from providers such as Amazon, Google, and Apple, and vary in shape, size, and cost, with some models having screens and cameras. For example, the Amazon Echo also contains a “drop-in” capability where devices, even if in different locations, can be linked and act as an intercom. Most also have the added capability of controlling appliances such as light bulbs, smart doorbells, and heating systems to create a “smart home.” Popular uses for speakers include music, information seeking, and entertainment [24,25], but they may extend to companionship, health care support, and better sleep [26]. Speech input and output provide increased accessibility for all users, but particularly for those with limited mobility and vision.

Hampshire and Oxfordshire County councils implemented Alexa devices for 60 people requiring support from social care [27,28]. Both pilot studies found improvements in service users’ ability to remain independent and feel less isolated. In Wales, the Innovate Trust investigated how smart speakers could meet the needs of adults with learning differences and reduce staff workload, thus saving money [29]. They estimated a potential saving of £20,000 (US $26,434) per year across two assisted living sites. There are an increasing number of studies examining the use of smart speakers among community-dwelling older adults [30], with qualitative feedback demonstrating commands on first interaction, which included asking for health care-related questions [31]. Although the results of a survey showed that half of care home staff think artificial intelligence in devices such as smart speakers should be in use to help care for residents [32], we found no evidence of the previous implementation of smart speakers in care homes at the majority of sites across the region under study.

The eHealth Productivity and Innovation in Cornwall and the Isles of Scilly (EPIC) project aims to develop the eHealth sector working with both the demand—improving the capability and capacity for using digital technologies—and the supply through supporting small companies producing new products and services. Early workshops identified loneliness as a major health problem and lack of skills in using technology in care homes as a barrier [33]. Voice technology was identified as an area where local expertise could be expanded, but it was clear that its use needed to be normalized [34], and a large user base would provide the incentive of a local market.

This study aimed therefore to address the loneliness and mental well-being of care home residents and at the same time to stimulate the improved uptake of technology in the care home sector, raising awareness and normalizing the use of video calls and voice-activated technologies among care home staff. We aimed to create an expectation of use across the sector.

We aimed to give at least one smart speaker device to 150 (65.2%) of 230 Cornish residential care homes and to explore if and how the devices were used, the barriers to their implementation, and their potential benefits.

## Methods

### Study Design

This mixed methods implementation study used care home staff diaries and telephone surveys to assess the use and impact of smart speakers. We took an eclectic approach to theory, with a lexicon borrowing ideas of “local demonstrability” [18], “technology acceptability” [16,17], implementation “at scale” [13,14], and “normalization” [15]. This eclectic conceptual framework drew mostly from NPT. The 4 NPT components were considered in our aim to understand if the devices were set up and used (cognitive participation), what they were used for (coherence), what barriers were experienced (collective action), and any potential benefits to residents and staff (reflexive monitoring). Ethical approval was granted by the Faculty of Health Ethics Committee (reference 18/19-1054).

### Choice of Smart Speakers

**Figure 1:**
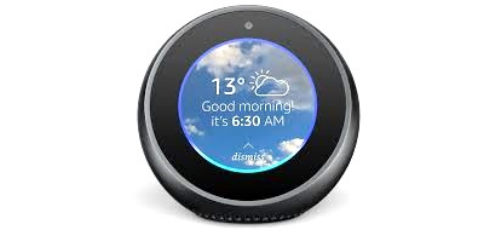
Amazon Echo Spot.

We aimed to implement devices that offered the possibility of video calls through a screen. In December 2018, the most appropriate device on the market was the Amazon Echo Spot (Figure 1; Multimedia Appendix 1). At Amazon’s suggestion, the Amazon Kindle Fire (Multimedia Appendix 1) was trialed in some homes. Early feedback indicated that Echo Spot devices were more physically robust, and Kindle Fires looked more like “regular tablets,” which some homes owned and had limited use for. With this feedback, Echo Spots were distributed until October 2019, when they were discontinued. Subsequently, we offered homes the Echo Show 5 (Multimedia Appendix 1).

### Care Homes

Using published lists [35] updated through local knowledge, 230 homes were identified. All homes were approached at least once, using a combination of the recruitment methods.

### Recruitment and Device Distribution

Initially, care homes were invited to 2 workshops, in West and East Cornwall. The participants were presented with project information and received written information, and those who agreed to participate were provided with a device. In total, 18 homes attended the 2 workshops, and 17 agreed to take part. Moreover, 4 further drop-in sessions were held over 5 months to enable care home staff to speak to researchers about the project. Those agreeing to participate were provided with a smart speaker. Drop-in sessions were advertised via postal and email campaigns and targeted telephone calling. Attendance at local drop-in sessions was low (6 people at 4 sessions from over 200 invited). However, all 6 agreed to participate. A substantial effort was also made via face-to-face recruitment with research assistants “cold calling” approximately 150 homes to explain the project and offer the opportunity to take a device. Cornwall Council also emailed all homes advertising the project resulting in 20 responses.

### Support Offered to Care Homes in Using the Smart Speaker

Ongoing support was offered to care homes by two methods. First, the core EPIC team, who had made the initial approach and supplied the device. A monthly email newsletter was circulated with suggestions on different ways to use the device. Second, Digital Health Champions were recruited via undergraduate nursing and occupational therapy programs and students aged 16 or more, from 2 secondary schools. Digital Health Champions were asked to support care home staff in using smart speakers. EPIC team members supported Digital Health Champions with guidance on how to use the device and monthly group Skype sessions as a group.

### Data Collection

Care homes were supplied with a short user guide and a diary to keep track of how and how often they were using the device, any barriers and how they were overcome, and any factors that enabled use of the device. Only 18 homes returned completed diaries; therefore, the remaining homes were contacted in early 2020 to take part in a short telephone survey to gain feedback on their device usage, their reason (if they were not using the device), and where it was located. We also asked for short descriptive accounts of their experiences including what the device was being used for and if they had experienced any problems. The responses were documented verbatim directly during the telephone call. Most (142, 95%) homes provided feedback either via diary or by telephone.

### Data Analysis

Survey data were analyzed using descriptive statistics. Descriptive experiences of the device usage, gathered from diaries and telephone survey, were qualitatively analyzed using thematic analysis. We followed the 6-step guide outlined by Braun and Clarke [36].

## Results

### Care Homes That Took 1 or More Devices

**Figure 2:**
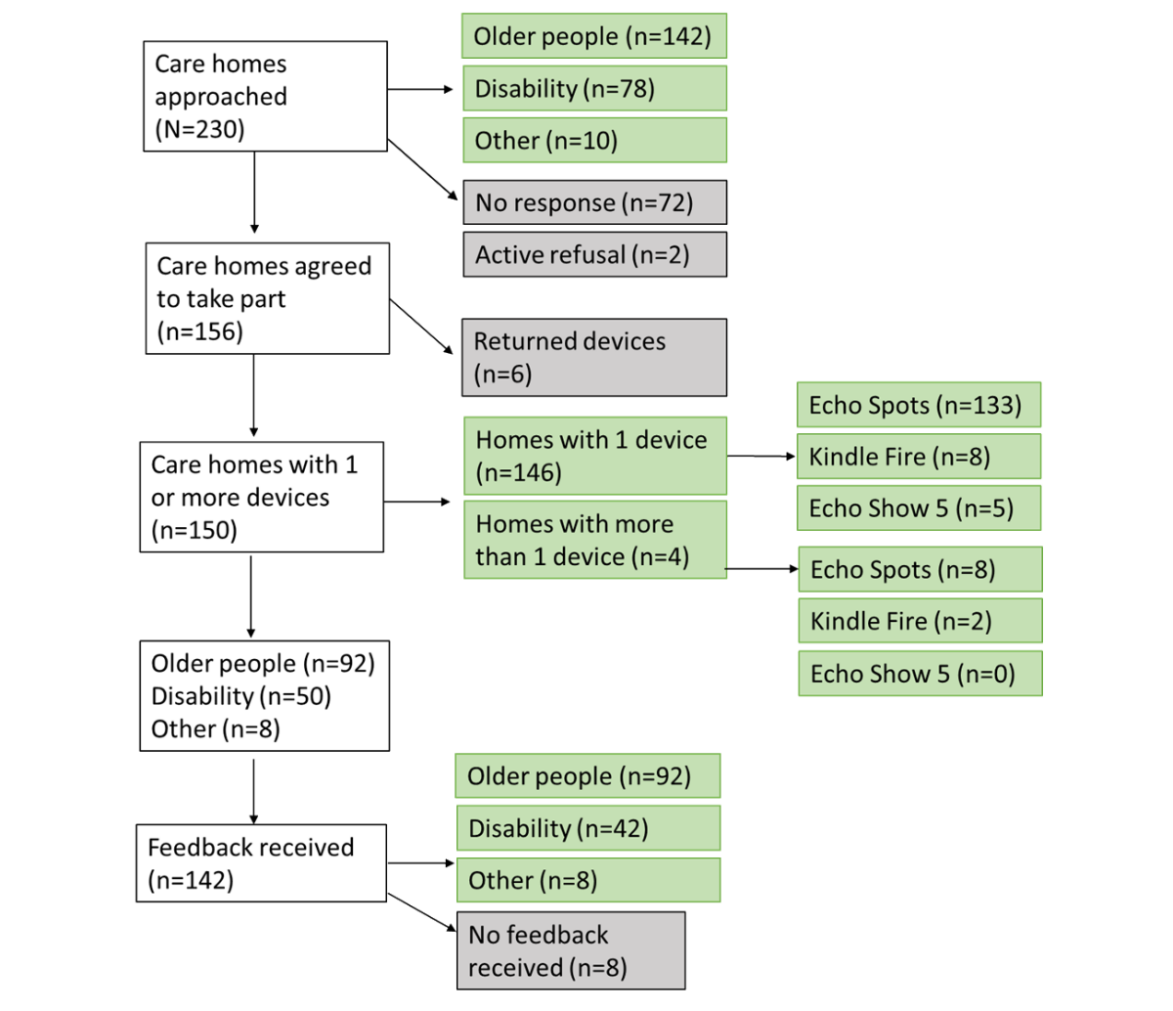
Flowchart of recruitment, interventions, and data collection.

**Figure 3:**
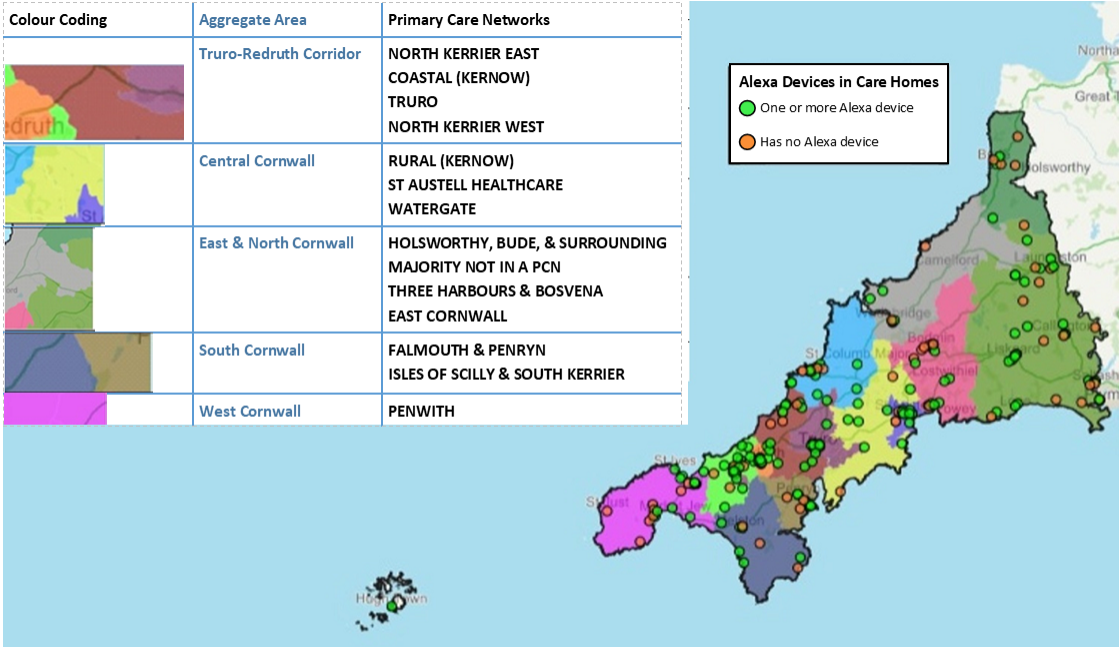
Location of 230 care homes in Cornwall and the Isles of Scilly showing 150 that accepted a smart speaker device and 80 that did not by primary care network areas. PCN: primary care network.

Of the 230 homes invited, 156 (68% initial uptake) homes took at least 1 device (Figure 2). Six homes returned their speaker, leaving 150 (65.2%) with devices (Figure 2). Dividing Cornwall into 5 areas (Figure 3) aggregating primary care networks, uptake was lower in East (44/79, 56%) and West Cornwall (12/23, 52%) compared with the other 3 areas (67%-78%; *X*^2^=10, 4 *df*; *P*=.04). The 150 homes with devices comprised 92 for older people (estimated resident population 2099 [24]) and 50 for those with physical or mental health needs (resident population 1097). Moreover, 10 homes received a Kindle Fire, 141 an Echo Spot, and 5 an Echo Show 5.

### Care Homes That Returned or Did Not Take a Device

The reasons for the 6 returned devices were as follows: (1) management concerns about access to confidential information; (2) staff thinking it was inappropriate for their clients; and (3) lack of internet access. Of the 72 homes (resident population 1740), not taking devices, 3 “positively” declined but 69 did not respond.

### Device Usage

Most (142, 95%) care homes provided feedback (Figure 2), of which three-quarters (107/142, 75%) were currently using the device (Table 1). More homes caring for older people were using the device than homes for people with physical and mental health needs (82% vs 61%; *X*^2^=7.1, 2 *df*; *P*=.03). Most homes (70/107, 65%) placed the device in communal areas such as living or dining rooms. Homes caring for older people were more likely to move the device around (26/75, 35%) compared with homes for people with physical or mental health needs (2/27, 7%) (Table 1).

**Table 1:** Care homes using one or more devices at follow-up, by care home type.

Home type and location of use		Homes, n (%)	Homes using the device, n (%)	Homes not using the device, n (%)	Total, n
**Older people**			75 (82)	16 (18)	91
	Moveable	26 (35)			
	Communal	44 (59)			
	Other	5 (7)			
**Physical or mental health needs**			27 (61)	17 (39)	44
	Moveable	2 (7)			
	Communal	22 (81)			
	Other	3 (11)			
**Other**			5 (71)	2 (29)	7
	Moveable	0 (0)			
	Communal	4 (80)			
	Other	1 (2)			
Total		N/A^a^	107 (75)	35 (25)	142

^a^N/A: not applicable.

### Thematic Analysis

We identified 5 themes and 26 subthemes, which can be seen in Multimedia Appendix 2.

### Usage

Virtually all (103/107, 96%) homes used devices for music and audio including listening to the radio, creating playlists, listening to Christmas carols, background music, and audiobooks (Multimedia Appendix 2). More than half (62/107, 58%) used it for information seeking, asking the speaker for the news, weather, and checking tide times when arranging walks. A third (35, 33%) used it for quizzes and games, 18 (17%) for jokes and comedy, and 4 (4%) for reminders. Eleven homes (10%) reported “general engagement” with residents talking to and engaging with the smart speaker. One manager reported “We have a resident who just loves to shout at Alexa, she gets in the neck but because she’s compliant, the resident loves it!” Video calling was reported by only 6 (6%) homes, although some homes expressed a desire to use it but had not yet tried. However, 2 (2%) homes were using screens as part of other activities (including watching videos), 8 (7%) in sing-along sessions, and 3 (3%) using the “drop-in” feature. One (1%) found the screen useful when running quizzes or adding enjoyment to listening to music.

Moreover, 4 (4%) homes reported “advanced use” such as connecting multiple devices to create a “call bell” for one bedbound resident; “We use it as a makeshift call button, which is linked to all the other devices; when the person presses the button, a song plays across the whole house, and we know he needs support.” One (1%) used the device to integrate with other smart technology.

### Benefits Associated With Use

In most homes (83/107, 78%) all residents had access to the device, 10 (9%) reported 75%-90% access, and 14 (13%) with more limited access. In diaries and telephone surveys, staff relayed benefits of using the speakers despite not being directly asked. These benefits were grouped into 5 themes (Multimedia Appendix 2). The most frequently reported benefit was resident enjoyment (36/107, 34%). For example, one reported, “the person we support has started to really enjoy music and will dance, relax, or feel vibrations on the echo spots speaker,” and another, “this has brought great joy and laughter to the home. Lots of dancing and singing currently taking place.”

Relaxation and calming were reported by 11 (10%) homes; for example, “when a client is suffering from agitation we asked for thunderstorms and the results where amazing relaxed the client almost within minutes and he slept peacefully.” Seven (7%) homes reported that the device offered companionship, one saying “Alexa is the resident’s companion that never sleeps, she is always there if a resident can’t sleep and wants some company.” Four (4%) homes reported the acquisition of a new skill set for residents as a benefit. One said “our resident has a speech difficulty, so struggles to ask it things, but it helps her to communicate more clearly. She sees staff using it and tries to copy.” The less frequently reported benefits were the time saved (2/107, 2%), increased staff engagement (3/107, 3%), and ease of use (2/107, 2%).

### Barriers Associated With Nonuse

Among the 35 care homes that reported not using the device, 15 (43%) belonged to a care provider whose IT specialist raised concerns about data protection (Multimedia Appendix 2). Other reported barriers included lack of time and resource (7/35, 20%), internet connectivity (4/35, 11%), and lack of skill or confidence around technology (6/35, 17%).

### Barriers Associated With Use

Most homes using their device (75/107, 70%) reported no barriers setting up and using the speaker. Homes who expressed issues most frequently reported internet connectivity problems (14/107, 13%). Nine (8%) homes reported device limitations including the smart speaker’s inability to understand certain residents because of speech difficulties or not using the “desired” language. Barriers to video calling included reluctance from family members and limitations associated with moving the device.

In 2 (2%) homes, residents had broken the device; however, both homes had repurchased devices and placed them in more secure locations. Four (4%) homes reported that the device caused confusion, or residents disliked it; however, in most cases, this was part of an adjustment period. One said, “I think originally they were a little suspicious of the speaker, but now they take it in their stride and just accept that they can ask this small ‘box’ questions!”

Data protection concerns were reported by 1 home with a device in use. Only 1 home reported resident inability to operate the device as a problem. A lack of skill or confidence in staff was reported by 1 home as a concern.

### Support Offered and Requested

Twenty (19%) homes contacted the core EPIC team between April 2019 and February 2020. Nine (8%) homes wanted support including with setup, internet connectivity, connecting two devices, or using Skype. Four (4%) homes requested an additional device, 1 wanted a device with a bigger screen, 1 to exchange their Fire tablet with a Spot, and 5 homes requested further support from a student to use the speaker more effectively.

Moreover, 24 university and 8 secondary school students became Digital Health Champions and borrowed a device. Each university student was assigned at least 2 care homes. Of the recruited care homes, 122 (81%) were linked with a Digital Health Champions and offered support via email, phone, or occasionally face-to-face. However, only 11 homes provided the students with feedback, despite numerous attempts to contact their care homes.

## Discussion

### Implementation at Scale and “Normalization” of Use

We aimed to get smart speaker devices used in 65% of care homes. This proved challenging but was achieved, benefitting residents and creating a “user base” of voice-activated devices. Although at follow-up 25% (35/142) were not yet using the device, redistribution and prompting was likely to bring these devices into use. Overall, there was emerging “normalization” [34] of smart speaker use in care homes in Cornwall.

### Uses and Benefits

Similar to pilot projects conducted in Oxfordshire and Hampshire [27,28], we received positive feedback on the use of smart speakers. Their use was similar to that in the general population [24,25], music and information-seeking being the most popular. While music is a basic function, its use supported opportunities for reminiscence; the speakers provided soothing music with end-of-life care, calming the residents.

Smart speakers may help reduce loneliness and increase independence [27,28]; 6 of our homes stated that the devices provided companionship for residents. Further exploration of functions that support independence, particularly for those living with physical or mental health needs, is required. For example, the “reminder” function has been shown to benefit individuals requiring support while living in their own homes [27,29,37], but only 4 of the 150 homes in our study reported spontaneous use of this function.

Similar to the findings by Pradhan et al [11], our results demonstrated unexpected uses, including 2 homes where the residents’ interaction with the smart speaker provided opportunities to practice and improve expressive language skills.

### Barriers to Implementation

Implementing new technology in care homes can prove challenging. Zamir et al [5] found that implementing video calls within care homes faced barriers such as staff turnover and lack of family commitment. In our study, only 6 homes reported using the devices to make video calls, suggesting that cheaper devices without screens could be used instead. However, screens were used intuitively as part of other activities including reading lyrics for sing-along sessions and displaying pictures to support information seeking. It remains to be seen how the COVID-19 pandemic has affected the use of video calls within care homes to overcome “shielding.”

Studies have cited the lack of digital literacy as a barrier to smart speaker use for individuals requiring social care support at home [27]. Only 1 home in our study that used a device reported a lack of confidence or skill as a barrier; however, for those not yet using it, a lack of skill among staff was a concern. One issue experienced by homes was insufficient internet connectivity; however, outages of Wi-Fi or main power did not pose an unacceptable inconvenience, nor did they seem to inhibit continued use. Changing working methods is always difficult [29], and undertaking activities to increase digital literacy of care home staff could facilitate adoption of voice-activated technology.

As Hoy [21] also found, privacy and data protection using smart speakers was of concern for a few homes and 1 home using the device at follow-up. Such concerns therefore appeared to inhibit adoption but did not limit use once installed. Others have found patient privacy and data protection concerns where there is more specific health care use [38], yet a pilot project in Stoke-on-Trent concluded that while concerns related to privacy and accessibility cannot be ignored, smart speakers may partially solve some problems facing primary care [39]. Our research suggests that smart speaker use in communal areas of care homes for mainly entertainment purposes creates few privacy concerns. Undertaking activities to increase digital literacy including knowledge of data security of care home staff could facilitate adoption of voice-activated technology for more health-related and care-related uses.

In our study, only 1 home refused to participate based on “not liking technology.“ However, others may find that cultural change hinders wider adoption. Changing working methods is always difficult, as found by a Welsh project on smart speakers in supported living [29]. Undertaking activities to increase digital literacy of care home staff could facilitate adoption of voice-activated technology.

### Negative Outcomes

None of the homes reported distress linked to the use of smart speakers, and only 4 homes reported temporary dislike or confusion by residents. However, confusion around the device did not typically affect enjoyment. Other reports suggest people with dementia have shown distress at having a robotic voice speaking to them [40]. How smart speakers are perceived and are of benefit to those experiencing dementia requires more in-depth exploration.

### Supporting Installation of New Technology

Although all care homes had the offer of help from a Digital Health Champion student, we had few (20/150) requests for support. In some cases, Digital Health Champion students were particularly active; but overall, this aspect of the project had limited uptake as students had difficulty contacting care homes, with most requests for help being received by the core research team. This may be due to the initial personal contact made for distributing the devices.

### Future Uses

As the use of smart speakers becomes normalized in care homes, one might expect an increased use for other purposes such as providing health care information or advice to staff. This extends the use beyond entertainment, and further research is needed to evaluate effective workflows [41]. Further research is also needed on designing voice user interfaces for health care, as current design training for voice interfaces is relatively limited [42].

### Limitations

The feedback collected was primarily from one person, usually the manager, and therefore may only represent a single stakeholder perspective. To gain more in-depth understanding of the practical use, barriers, and facilitators of smart speakers, further research is needed from a broader stakeholder base including residents, families, and the Digital Health Champions. On collection of feedback, homes were not specifically asked to share the benefits of device usage; therefore, the benefits experienced may be understated. We had hoped that care homes would keep diaries and be able to tell us how many of their residents interacted with the smart speakers, but this data collection proved impractical. Systematic exploration of benefits and how they related to specific smart speaker “skills” and subsets of service users will be important for future research. Further exploration of the sustainability of benefits of the smart speakers is required in the longer term beyond 3-6 months.

### Conclusions

This study demonstrated that most care homes are prepared to install and use smart speakers to benefit staff and residents. As an affordable and readily available commercial product, smart speakers represent a highly scalable option to facilitate technology-enabled care. Future work needs to explore how to reach the remaining care homes, deal with cybersecurity concerns, highlight beneficial “skills” for residents in the longer term, and investigate the impact on staff workload. This may lead to opportunities for smart speaker software development supported by local small and midsize enterprises.

## Appendix

Multimedia Appendix 1 Amazon Echo Spot, Kindle Fire, and Echo Show 5.

Multimedia Appendix 2 Themes, sub-themes, and example quotes.

## Abbreviations

EPIC: eHealth Productivity and Innovation in Cornwall and the Isles of Scilly
NPT: Normalization Process Theory
