# Supplementary figures and images for: The Use of Smart Speakers in Care Home Residents: Implementation Study
Source: J Med Internet Res. 2021 Dec 20;23(12):e26767. doi: 10.2196/26767 (PMC8726051; doi:10.2196/26767)

**Appendix I**

Amazon Echo spot


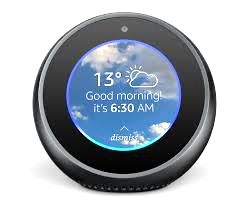


Amazon Kindle Fire


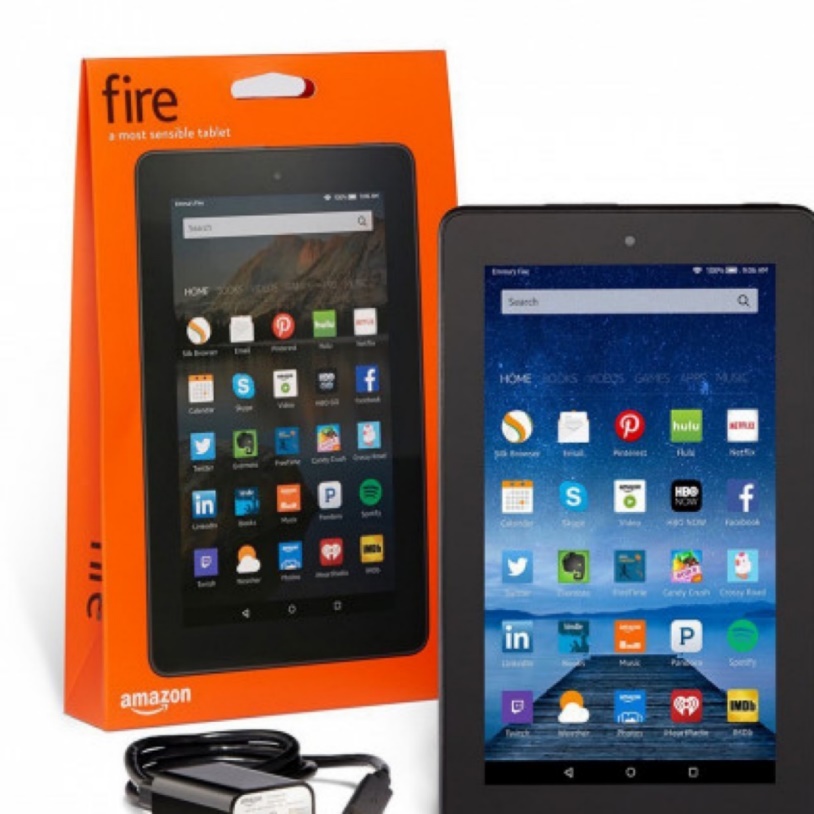


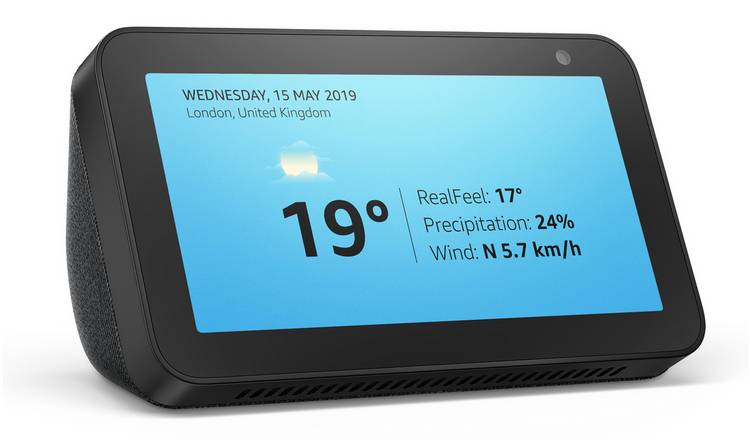
Amazon Echo Show 5

Supplement: Multimedia Appendix 1 [file jmir_v23i12e26767_app1.docx]
