# Supplementary material for: The Use of Smart Speakers in Care Home Residents: Implementation Study
Source: J Med Internet Res. 2021 Dec 20;23(12):e26767. doi: 10.2196/26767 (PMC8726051; doi:10.2196/26767)
Supplement: Multimedia Appendix 2 [file jmir_v23i12e26767_app2.docx]

| Themes and Subthemes | | Example quotes |
| --- | --- | --- |
| **Usage** | | |
|  | - Music and audio - Quizzes and games - Jokes and comedy - Information seeking - Screen use - Reminders - General engagement or chat - Advanced Uses | - It has been a massive hit with our residents, and we have gone on to buy 2 Echo dots. We have downloaded audio books and created playlists for individual residents, and they are really enjoying being able to listen to whatever they fancy. - Skyping families in the USA and Australia has made a real difference to residents and families. - Our residents chat to it, say good morning and good night to it. |
| **Benefits associated with use** | | |
|  | - Staff focused benefits - Resident enjoyment - Resident engagement - Technology enabled care - Unexpected benefits - Companionship | - Gentlemen on bed rest who is unable to participate in activities. Put on free audible book [sic] for him. He really enjoyed listening and was engaged for several hours, calmly listening to the book. Fantastic option for someone who has poor eyesight and is unable to leave bed. |
| **Barriers associated with use** | | |
|  | - Video calling - Internet connectivity - Limitation with device - Resident focused problem - Staff focused problem - Data protection | - Sometimes residents video call the other building but family members haven’t been keen on the video calling, but they are quite elderly themselves. |
| **Barriers associated with nonuse** | | |
|  | - Internet connectivity - Limitation with device - Resident-focused barriers - Staff-focused barriers - Data protection | - We have become mired around finance, capacity, best interests, and to top it all, needing to have parallel wireless networks in each home, some we have a corporate network for work devices but not guest, we do not want random devices on the secure network as it can access a whole host of other things. |
| **Support requested** | | |
|  | - Support | - You asked if we needed any assistance, the only thing I could think of is how to communicate between the two speakers, is that possible? It might be useful for the resident in her room to talk to me in the lounge. |
